# The prevalence and determinants of social anxiety disorder among people living with HIV/AIDS in Southwestern Ethiopia: a cross-sectional study

**DOI:** 10.3389/fpsyt.2024.1437891

**Published:** 2024-10-04

**Authors:** Yadeta Alemayehu, Mustefa Adem Hussen, Zakir Abdu, Aman Dule, Mohammedamin Hajure, Gebremeskel Mulatu, Wubishet Gezimu, Solomon Seyife Alemu, Lema Fikadu Wedajo

**Affiliations:** ^1^ Psychiatry Department, College of Health Sciences, Mattu University, Mattu, Ethiopia; ^2^ Midwifery Department, College of Health Sciences, Mattu University, Mattu, Ethiopia; ^3^ Department of Psychiatry, Maddawalabu University, Shashemene, Ethiopia; ^4^ Department of Nursing, College of Health Sciences, Mattu University, Mattu, Ethiopia; ^5^ Midwifery Department, Maddawalabu University, Shashemene, Ethiopia; ^6^ Midwifery Department, College of Health Sciences, Wallaga University, Nekemte, Ethiopia

**Keywords:** social anxiety disorder, HIV/AIDS, Ethiopia, prevalence, determinants

## Abstract

**Background:**

Social anxiety disorder imposes impacts of functional disability, poor educational achievement, loss of work productivity, social impairment, greater financial dependency, and impairment in quality of life. Therefore, the aim of this study was to assess the prevalence and identify determinants of social anxiety disorder among people living with HIV/AIDS.

**Methods:**

A cross-sectional study was conducted from 1 to 30 May 2022 among 354 people living with HIV using a simple random sampling technique. The Social Phobia Inventory (SPIN) Scale was used to assess the presence of social anxiety disorder. Data were gathered using chart review tools and a structured questionnaire, which was administered by a pretested face-to-face interviewer. SPSS version 25 was used to analyse the data once they were imported into EpiData Manager version 4.6. Binary and multivariable logistic regressions were performed. After calculating odds ratios with a 95% confidence interval (CI), statistical significance was established at *p* < 0.05.

**Results:**

A total of 336 respondents participated in the study, resulting in a response rate of 94.91%. The magnitude of social anxiety disorder was 32.44% (95% CI: 27.4, 37.2). Being female [adjusted odds ratio (AOR) = 3.55, 95% CI: 1.61, 7.84], having a stage III/IV HIV/AIDS status (AOR = 3.17, 95% CI: 1.10, 9.13), being alcohol dependent (AOR = 2.81, 95% CI: 1.45, 5.44), and having perceived stigma (AOR = 5.62, 95% CI: 2.95, 10.72) were predictors of social anxiety disorder.

**Conclusion:**

In this study, approximately one-third of people living with HIV/AIDS had social anxiety disorder. Being female, having a stage III/IV HIV/AIDS status, being alcohol dependent, and having perceived stigma were predictors of social anxiety disorder. Therefore, training for health care providers on the screening, counselling, and management of social anxiety disorder is important.

## Introduction

A persistent fear of one or more social situations (interactions, observations, and performances) in which embarrassment may occur is known as social anxiety disorder or social phobia. Fear and anxiety are disproportionate to the actual threat posed by the social situation, which is determined by the person’s cultural norms like eating and drinking in public (1, 2). People with HIV often report feeling anxious and fearful (3). Whether diagnosed early or late with HIV infection, anxiety is a common emotional response observed in people living with HIV/AIDS (PLWHA) (4). People who experience excessive anxiety may feel powerless in the present or against themselves. They may continue their patterns of inappropriate behaviour in the future (5).

In general, excessive or irrational anxiety reactions, together with behavioural and cognitive reactions, clinically significant distress, and dysfunction, are features of social anxiety disorder (2). Significant consequences of social anxiety disorder are observed in people with the disorder and their families and caregivers, as well as society in general, due to functional impairment, poor academic performance, decreased productivity, social impairment, increased financial dependence, and a decreased quality of life (5, 6).

Approximately 20.6 million people in eastern and southern Africa are HIV positive, and AIDS-related mortality accounted for 0.28 million deaths in this region (7). The adult (15-49 age) HIV prevalence in Ethiopia is 0.93% across the country, with Gambela having the highest rate at 4.45% and Somali regions having the lowest rate at 0.16%. The prevalences in urban areas, rural areas, females, and males are 2.9%, 0.4%, 1.2%, and 0.7%, respectively (8, 9).

Compared with the general population, PLWHA are more likely to suffer from social anxiety disorders (10). According to recent studies, the prevalence of social anxiety disorder among people with HIV was 70.44% in Brazil (11), 27% in China (12), and 32.4% in Ethiopia (13).

As they cope with the diagnosis of HIV/AIDS and deal with the challenges of having a chronic life-threatening illness—such as reduced life expectancy, challenging treatment plans, stigma in various forms, and losing the support of friends and family—people living with the virus frequently experience anxiety (12, 13). Studies have shown that people with HIV/AIDS are more likely to experience mental health problems, such as anxiety (14, 15). As a result, these conditions may weaken their immune systems, reduce their quality of life and adherence to therapy, and eventually cause early death (11, 16). Before HIV infection, anxiety may be present, or anxiety may be linked to risky behaviours for HIV infection, such as drug use or unsafe sexual activity (4). Additionally, people may experience anxiety during or after their diagnosis due to the possibility of an undiagnosed HIV-related mortality (17). Social anxiety disorder and antiretroviral therapy (ART) non-adherence are significantly correlated (11, 18).

One of the sustainable development goals is to end the HIV epidemic; therefore, studying social anxiety disorder in PLWHA makes sense. There are, however, few data on social anxiety disorder in Ethiopia. Drawing on current realistic research, it is plausible to speculate that social anxiety disorders and their symptoms could influence an individual’s decision to either engage in or abstain from behaviours that increase their risk of HIV/AIDS, such as drug use and unprotected sexual activity. Additionally, anxiety psychopathology could be connected to an individual’s lived experience with the virus. Considering the co-occurrence of social anxiety disorder and HIV/AIDS, it is essential to create integrated intervention strategies for the prevention, management, and enhancement of both conditions. Gaining a deeper comprehension of the relationship between HIV/AIDS and social anxiety disorders would help researchers address the issue by improving our knowledge of the mechanisms behind the co-occurring mental illnesses and the negative effects these conditions have on overall health. Therefore, the purpose of the current research was to assess the prevalence and identify determinants of social anxiety disorder among PLWHA in Southwest Ethiopia.

## Methods and materials

### Study design and period

A cross-sectional study design was conducted among PLWHA at the ART clinic of Mattu Karl Comprehensive Specialized Hospital, Mattu, Oromia regional state, Ethiopia, from 1 to 30 May 2022.

### Study setting

Mattu Karl Comprehensive Specialized Hospital (MKCSH) is found in Mattu City in Oromia Regional state of Ethiopia. The hospital provides services such as surgery, gynaecology, obstetrics, medicine, paediatrics, an outpatient department, diagnostic facilities, an ART clinic, psychiatry, and intensive care. Currently, 1,740 people are taking ART at the hospital.

### Study participants

The source population consisted of all people with an HIV/AIDS diagnosis attending an ART clinic at MKCSH. The study population consisted of randomly selected individuals living with HIV/AIDS who were attending the hospital’s ART clinic during the data collecting period. PLWHA who were 18 years of age or older and enrolled in a hospital ART clinic throughout the time of data collection for ART services were included. Those PLWHA who were seriously ill and failed to communicate were excluded.

### Sample size determination

The sample size required for the study was calculated using a single population proportion formula by considering the prevalence rate of social anxiety disorder among PLWHA of 32.4% (13), a 5% margin of error, a 95% level of confidence interval, and a 5% non-response rate. When taking the 5% non-response rate into account, the final calculated sample size of the study was 354.

### Sampling procedure

The total number of PLWHA was obtained from the record of the registration book available at the ART clinic of MKCSH. The recorded medical registration numbers were arranged in ascending order to form a frame. To identify study participants, a random number generator in SPSS was used to build the frame. Finally, once the desired sample size was obtained, the research participants were chosen at random from the constructed frame using a straightforward random sampling procedure.

### Data collection procedures and tools

Data were collected using a pretested structured questionnaire and the face-to-face interview technique. Initially, the questionnaire prepared in the English language was translated to Amharic and Afan Oromo and then translated back to English by language experts to check the consistency and understandability of the tools. The tools contained sociodemographic characteristics, medical information related to HIV/AIDS, social support, and social anxiety disorder. Social anxiety disorder was assessed using the Social Phobia Inventory (SPIN), which is a 17-item self-rating scale rated from 0 (not at all) to 4 (extremely), and the sum score ranged from 0 to 68, with a score of 20 and above on the SPIN showing the presence of social anxiety disorder (19, 20). The SPIN results had a Cronbach α = 0.93 in the current study. The Oslo Social Support Scale (OSS-3) and the HIV perceived stigma scale were used to measure social support and stigma in PLWHA, respectively (21, 22). In the current study, the OSS-3 and HIV perceived stigma scale had a Cronbach α = 0.91 and α = 0.89, respectively. The medical data related to HIV/AIDS, such as comorbidities, CD4 count, ART, and infection duration, were collected from medical records. The proportion of current substance use and lifetime substance use were considered when participants had used at least one of the specified substances in the last 3 months and used at least one of the specified substances in their lifetime, respectively, by using the adopted alcohol, smoking, and substance involvement screening test (23).

### Operational definition

Social anxiety disorder: a total sum of 20 or greater on the SPIN scale indicates social anxiety disorder (19, 20).

Social support: the OSS-3, which is a three-item scale exploring the number of close friends, perceived level of concern from others, and perceived ease of getting help from neighbours, was used to assess the level of social support. Scores of 3–8, 9–11, and 12–14 were considered as a low, medium, and high level of social support, respectively (21).

Perceived stigma: Individuals who scored >26 on thirteen items of the perceived stigma scale (22).

### Data processing and analysis

The obtained data were coded, verified for accuracy, and added to Epidata version 4.6. The data were imported and analysed using SPSS version 25.0. The outcome was addressed in relation to earlier findings and displayed in frequency tables. To determine the participants’ demographics, descriptive statistics, including mean with standard deviation, frequencies, and percentages, were employed. Additionally, the significance of the association was calculated using bivariable logistic regression analysis. Strongly correlated variables have *p*-values less than 0.05. The adjusted odds ratio (AOR) with a 95% confidence interval was used to describe the significance of the connection between the variables.

## Result

### Sociodemographic data

With 336 participants in all, the study had a 94.91% response rate. Of the responders, 137 (40.80%) were in the 25–34 age range and 185 (55.10%) were men. Approximately half of the participants (169, or 50.30%) were married, and 146 (43.50%) were Orthodox Christians. Over three-quarters of the respondents (130, 38.70%) only attended primary school and 152 (45.20%) did not have a job (**Table 1**).

**Table 1:** Sociodemographic characteristics of people living with HIV/AIDS at the ART clinic of Mattu Karl Comprehensive Specialized Hospital, Mattu, Oromia regional state, Ethiopia, 2022 (n=336).

Variables	Category	Frequency	Percentage (%)
Sex	Male	185	55.10
	Female	151	44.90
Age	18–24	58	17.30
	25–34	137	40.80
	35–44	96	28.60
	≥45	45	13.40
Religion	Orthodox	146	43.50
	Protestant	81	24.10
	Muslim	109	32.40
Ethnicity	Oromo	198	58.90
	Amhara	87	25.90
	Others*	51	15.20
Marital status	Married	169	50.30
	Single	72	21.43
	Divorced/widowed	95	28.27
Educational status	Unable to read and write	67	19.90
	1–8	130	38.70
	9–12	116	34.50
	Diploma and above	23	6.80
Type of occupation	Farmer	44	13.10
	Private work	66	19.60
	Government employee	21	6.30
	Driver	15	4.50
	Others	31	9.20
	Jobless	152	45.20
Living arrangement	With family	271	80.70
	Alone	65	19.30
Residence	Urban	270	80.40
	Rural	66	19.60

'*' indicates 'Tigre, Nuer, Anuak, and Gurage'.

### Clinical and behavioural factors

Of the participants, 89 (26.5%) were dependent on alcohol and 112 (33.3%) had used tobacco. The majority of responders (219, or 65.18%) had stage I HIV/AIDS. Approximately one-third (30.65%) of them were thought to be heavily stigmatised (**Table 2**).

**Table 2:** Description of the clinical and psychosocial factors of the respondents with HIV/AIDS at the ART clinic of Mattu Karl Comprehensive Specialized Hospital, Mattu, Oromia regional state, Ethiopia, 2022 (n=336).

Variables	Category	Frequency	Percentage (%)
CD4 count	=<500 cells/ml	142	42.26
	>500 cells/ml	194	57.74
Stage of HIV/AIDS	Stage I	219	65.18
	Stage II	93	27.68
	Stage III and IV	24	7.14
Duration of treatment	<5 years	159	47.32
	5–10 years	141	41.97
	≥10 years	36	10.71
Alcohol use	Yes	148	44.00
	No	188	56.00
Alcohol dependence	Yes	89	26.50
	No	247	73.50
Tobacco use	Yes	112	33.33
	No	224	66.67
Perceived stigma	High	103	30.65
	Low	233	69.35

### Magnitude of social anxiety disorder

**Figure 1:**
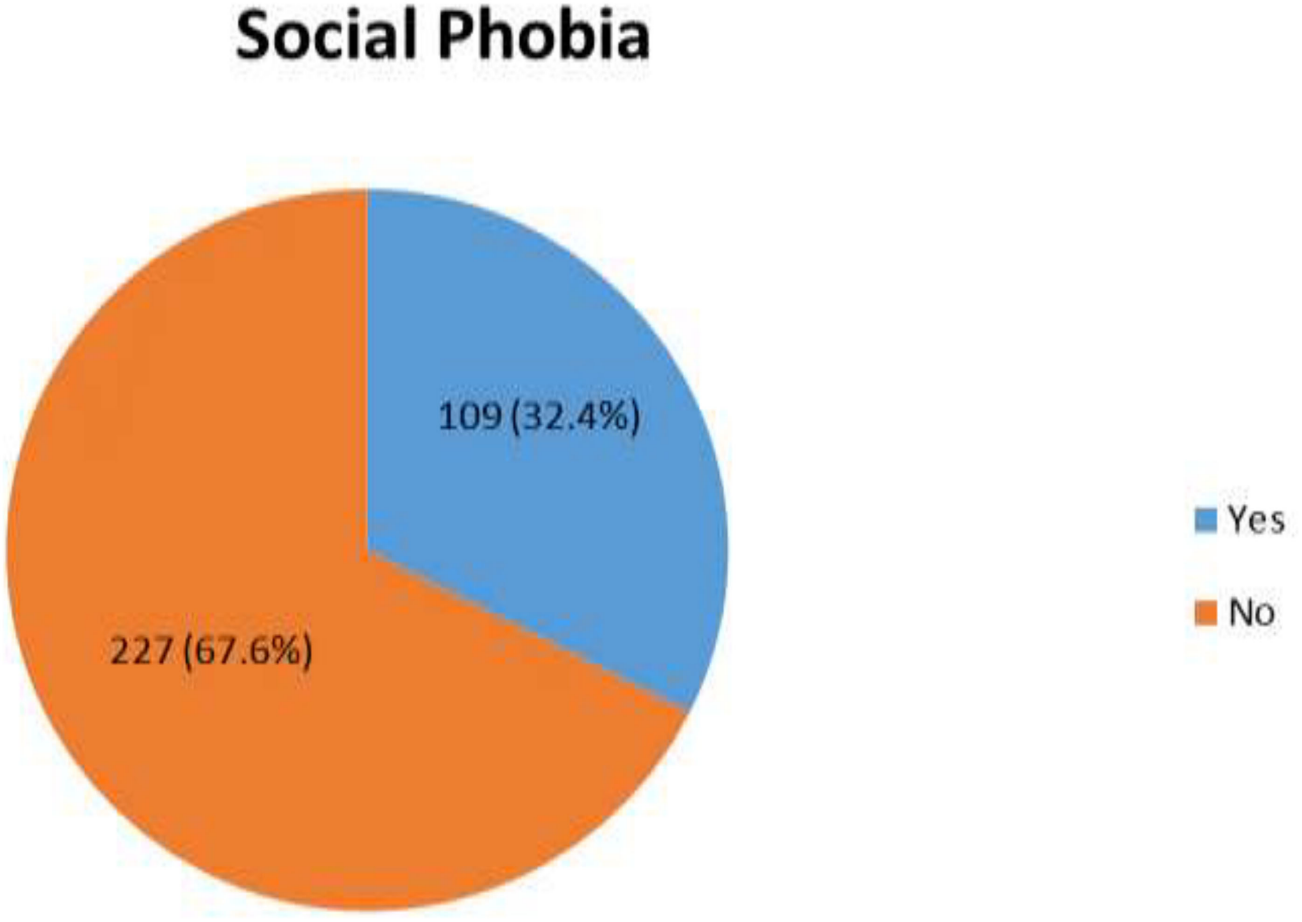
The magnitude of social anxiety disorder among respondents with HIV/AIDS at the ART clinic of Mattu Karl Comprehensive Specialized Hospital, Mattu, Oromia regional state, Ethiopia, 2022 (n=336).

Among the respondents, 109 (32.4%) had social anxiety disorder (95% CI: 27.4, 37.2) (**Figure 1**).

### Factors associated with social anxiety disorder

Among the variables used in this study, being female, being 35 to 44 years of age, being single, having stage III/IV HIV/AIDS, living alone, having alcohol dependence, and having high perceived stigma were considered in bivariate binary logistic regression with a *p*-value of less than 0.2. In the final model, being female, having stage III/IV HIV/AIDS, having alcohol dependence, and having high perceived stigma had a statistically significant association with social anxiety disorder (*p*-value less than 0.05).

The odds of having social anxiety disorder among respondents who were female were 3.55 times higher than for those who were male (AOR=3.55, 95% CI: 1.61, 7.84). Similarly, the odds of having social anxiety disorder among respondents who had a stage III/IV HIV/AIDS status were 3.17 times higher than for those who had a stage I HIV/AIDS status (AOR=3.17, 95% CI: 1.10, 9.13). Furthermore, the odds of having social anxiety disorder among respondents who were alcohol dependent were 2.81 times higher than for those who were on not alcohol dependent (AOR=2.81, 95% CI: 1.45, 5.44). Moreover, the odds of having social anxiety disorder among respondents who had high perceived stigma were 5.62 times higher than for those who had low perceived stigma (AOR=5.62, 95% CI: 2.95, 10.72) (**Table 3**).

**Table 3:** Bivariate and multivariate logistic regression analysis showing an association between social anxiety disorder and explanatory variables among people living with HIV/AIDS at the ART clinic of Mattu Karl Comprehensive Specialized Hospital, Mattu, Oromia regional state, Ethiopia, 2022 (n=336).

Explanatory variables	Social anxiety disorder		COR (95% CI)	AOR (95% CI)	*p*-value
	Yes	No			
Sex					
Female Male	74 35	77 150	4.12 (2.53, 6.70) 1	**3.55 (1.61, 7.84)** 1	**0.002**
Age					
18–24 25–34 35–44 ≥45	27 53 16 13	31 84 80 32	1 0.72 (0.39, 1.35) 0.23 (0.11, 0.48) 0.47 (0.20, 1.07)	1 1.27 (0.40, 4.02) 1.01 (0.25, 3.99) 1.77 (0.36, 8.68)	0.149 0.172 0.087
Marital status					
Married Single Divorced/widowed	30 41 38	42 128 57	1 0.45 (0.25,0.81) 0.93 (0.50,1.74)	1 0.59 (0.21, 1.70) 1.30 (0.41, 4.13)	0.163 0.115
Stage of HIV/AIDS					
Stage I Stage II Stage III/IV	67 29 13	152 64 11	1 1.03 (0.61,1.74) 2.68 (1.14,6.29)	1 0.96 (0.51, 1.83) **3.17 (1.10, 9.13)**	0.076 **0.033**
Alcohol dependence					
Yes No	37 72	52 175	1.73 (1.05, 2.86) 1	**2.81 (1.45, 5.44)** **1**	**0.002**
Living arrangement					
With family Alone	78 31	193 34	1 2.26 (1.30, 3.92)	1 0.57 (0.27, 1.23)	0.089
Perceived stigma					
High Low	66 43	37 190	7.88 (4.68,13.27) 1	**5.62 (2.95, 10.72)** 1	**0.001**

NB, A p-value of less than 0.05 is considered statistically significant. AOR, adjusted odds ratio; COR, crude odds ratio.The bold values in this table indicate "statistically significant variables".

## Discussion

The current study showed that the prevalence of social anxiety disorder was 32.5% (95% CI: 27.4, 37.2). The magnitude of social anxiety disorder in this study was in line with previous studies: 32.4% in Ethiopia (13), 28.9 in another study in Ethiopia (24), 32.6% in Nigeria (25), and 28.9% in the USA (26).

The prevalence was lower than in studies conducted in Ethiopia [39.2% (27)] and Ghana [61.4% (28)]. The possible reason for the variation could be that the studies used different tools for the assessment of the outcome variable. The study in Ethiopia used the Beck Anxiety Inventory (BAI-II) tool and the study in Ghana used the Hospital Anxiety and Depression Scale (HADS) assessment tool, which vary from the SPIN tool used in the current study. Additionally, the study in Ethiopia involved all clients who attended voluntary counselling and the testing centre (27), and the study in Ghana included only those participants aged ≥30 years (28).

The frequency was greater than research from Ethiopia, which indicates 25.6% (29), 17.4% (30), 22.2% (15); in Australia 22.9 (31) and 7% in California (32). The possible reason for the variation could be the difference in assessment tools and population character. The studies conducted in Ethiopia used the hHADS and BAI-II, which assess general anxiety rather than social anxiety disorder unlike the SPIN tool used in the current study. The study in Australia included only male patients and the study in California, USA, included all patients on primary health care. Therefore, as being female is associated with anxiety in different studies, including the current study, those that included only male patients may tend to have a lower magnitude (15, 29, 30).

Compared with men, women are 3.55 times more likely to suffer from social anxiety disorder. This is supported by the studies conducted in Ethiopia (15, 29, 30) and Nigeria (25). The possible interpretation could be the hormonal fluctuations in females related to puberty, the pre-menstrual period, pregnancy or postpartum, and the menopausal transition (33, 34). The other possible reason could be a greater sensitivity of females to stressful and traumatic life experiences (35).

Those with a stage III/IV HIV/AIDS status are 3.17 times more likely to have social anxiety disorder than those with a stage I HIV/AIDS status. This is supported by the study conducted in Ethiopia (4). The possible reason could be those patients at stages III and IV tend to have a symptomatic manifestation of HIV/AIDS, which makes them fear being involving in different social activities. Studies also show that as the severity of illness increases, the magnitude of neuropsychiatric problems such as social anxiety disorder increases (36, 37).

Respondents who were alcohol dependent had higher levels of social anxiety disorder than their counterparts. This is supported by the study conducted in Ghana (28). The possible interpretation for this association could be their expectation that alcohol will reduce anxiety in social situations and therefore they use it as a form of self-medication to reduce their fear (38, 39).

Participants who have high perceived stigma are more likely to have social anxiety disorder. This is demonstrated by the studies conducted in Ethiopia (4, 15, 29), Kenya (40) and the USA (41). The possible reason for this association might be those who perceive stigma tend to have less friends and isolate themselves from society, thus limiting social interactions, which leads an individual to develop a fear of being judged by others and being embarrassed about their actions (42).

A limitation of the study is that it used a cross-sectional study design, which fails to show temporal relationships between associated factors and social anxiety disorder. In addition, the finding of this study might be affected by social desirability bias.

## Conclusion

In this study, approximately one-third (32.4%) of PLWHA had social anxiety disorder. The prevalence of social anxiety disorder in this study was higher than in the majority of other studies. Among the explanatory variables, being female, having stage III/IV HIV/AIDS, being alcohol dependent, and having high perceived stigma were shown to have a significant association with social anxiety disorder. Therefore, routine screening for social anxiety disorder at ART clinics is important for providing holistic care for PLWHA. In addition, concern should be given to tackling stigmas felt by patients and routinely assessing the use of alcohol during follow up. The governing body is also expected to develop a programme and policy that help to minimise problematic fear and stigma among PLWHA.

## Data Availability

The original contributions presented in the study are included in the article/supplementary material. Further inquiries can be directed to the corresponding author.
